# Milk for Infants

**Published:** 1890-02-22

**Authors:** 


### DIETETICS.

### MILK FOR INFANTS.

We have already noticed ("Retrospect," Jan. 25) Dr. Burney Yeo's remarks on the diet of adults, and it now remains to epitomise his observations on the feeding of children. Most authorities, Dr. Burney Yeo says, are agreed as to the date at which food, other than milk, may wisely be introduced into an infant's dietary. Farinaceous food ought never to be given before the fourth month, and better not until after the seventh or eighth, when the teeth and salivary glands begin to be developed. Any of the malted foods may be usefully introduced at this period. Between the tenth and twelfth months breast feeding may be gradually suspended, and at this date a little meat may wisely be given. The chief difficulty in supplying a suitable substitute for human milk appears to depend, not so much on differences in the quantity of the various constituents, but on difference in the quality, especially of the albuminates. All artificial food should, like human milk, clot in fine flakes, therefore cow's milk has to be modified in preparation. To do this we have to dilute with water to bring the casein down to a proper level; to add cream in order to bring the proportion of fat up to the proper level; and to add sugar of milk in order to bring the proportion of sugar to the proper level. Much difference of opinion prevails as to the utility of condensed milk, Ashby and Wright doubting much the value of the evidence which has been brought against it. It is undoubtedly convenient, and may be substituted with good effect for cows' milk, when the latter, as sometimes happens, disagrees. The deficiency of casein may be supplied by the addition of a little white of egg, and the addition of a little cream will supply any deficiency in fat. Now, in our opinion, when properly diluted cow-milk (by which we mean from one-half to one-third water) disagrees, it will often be found that the cause is bad feeding of the animal. It is well-known that food makes great differences in human milk. Frequently a poor, ill-fed, wet nurse, suddenly transplanted into a wealthy family, and fed in comparison luxuriously, secretes milk which does not agree with the child; and it is well known that certain articles of diet influence the milk. It is the same with animals. Cows fed on turnips or grains often secrete milk which does not agree with children. So after a season of draught, when rain falls and young grass springs up, cows consuming such grass may produce milk with laxative properties. The child then suffer from diarrhœa, and medicines are given in vain while the exciting cause remains in operation. Again, there is another fertile cause of milk not agreeing. It is apt to become soured in hot weather, and the slightest approach to this condition is sufficient to disorder the infantile stomach. Feeding-bottles imperfectly cleaned must not be forgotton. Milk is often said not to agree when the reason is that some particles of decomposed milk lurk about the tube, bottle and cork, or whatever appliance may be used. If the greatest security is to be attained an animal should be kept expressly for the use of the child, and the feeding of the animal should be closely watched. The milk of two animals should never be mixed. Fresh-drawn milk should always be used, and the greatest care as regards the cleanliness of the bottle, &c., should be enforced. We can scarcely be surprised that the milk brought to the door in cans, the product of various animals, and possibly not altogether in good condition, does not always agree with infants.

Dr. Burney Yeo further tells us that pre-digested foods are of great value in certain troublesome cases—partially peptonised foods—the process being arrested before any distinctly bitter taste becomes developed. They should not, however, be continued longer than necessary, as the digestive organs require to be exercised in order to be healthily developed, as is the case with all the other organs of the body.

